# *Agrimonia eupatoria* L. and *Cynara cardunculus* L. Water Infusions: Comparison of Anti-Diabetic Activities

**DOI:** 10.3390/molecules21050564

**Published:** 2016-04-28

**Authors:** Anika Kuczmannová, Andrea Balažová, Eva Račanská, Miroslava Kameníková, Silvia Fialová, Jaroslav Majerník, Milan Nagy, Peter Gál, Pavel Mučaji

**Affiliations:** 1 Department of Pharmacognosy and Botany, Faculty of Pharmacy, Comenius University, Odbojárov 10, Bratislava 83232, Slovakia; kuczmannova@fpharm.uniba.sk (A.K.); kamenikova@fpharm.uniba.sk (M.K.); fialova@fpharm.uniba.sk (S.F.); nagy@fpharm.uniba.sk (M.N.); 2 Department of Cell and Molecular Biology of Drugs, Faculty of Pharmacy, Comenius University, Kalinčiakova 8, Bratislava 83232, Slovakia; balazova@fpharm.uniba.sk; 3 Department of Pharmacology and Toxicology, Faculty of Pharmacy, Comenius University, Odbojárov 10, Bratislava 83232, Slovakia; racanska@fpharm.uniba.sk; 4 Department of Medical Informatics, Faculty of Medicine, Pavol Jozef Šafárik University, Trieda SNP 1, Košice 04011, Slovakia; jaroslav.majernik@upjs.sk; 5 Department of Pharmacology, Faculty of Medicine, Pavol Jozef Šafárik University, Trieda SNP 1, Košice 04011, Slovakia; 6 Department for Biomedical Research, East-Slovak Institute of Cardiovascular Diseases, Inc., Ondavská 8, Košice 04011, Slovakia

**Keywords:** diabetes mellitus, phytotherapy, agrimony, artichoke, streptozotocin

## Abstract

Diabetes mellitus (DM) is frequently diagnosed at a time when patients already suffer from several cardiovascular complications. Our previously published data (Molecules 201520 (11): 20538-50) on the anti-oxidative properties of *Agrimonia eupatoria* L. (AE) and *Cynara cardunculus* L. (CC) prompted us to extend the available evidence on their possible protective activities on selected DM-related parameters in a streptozotocin-induced DM rat model and in a series of *in vitro* experiments. Male rats were divided into four groups: control group, untreated diabetic group, AE and CC treated diabetic groups. During a five-week period, changes in blood glucose and body weight were monitored. Then, rats were sacrificed and subjected to the assessment of changes in the reactivity of aortas and measurement of butyrylcholinesterase activity. To complete the panel of experiments, α-glucosidase activity was assessed *in vitro*. Our results demonstrate that both tested extracts exert similar anti-diabetic activities. However, better anti-oxidant activity of the *A. eupatoria* extract indicates its higher clinical potential in the prevention and/or adjuvant therapy of developing cardiovascular complications related to DM and diseases associated with oxidative stress.

## 1. Introduction

Diabetes mellitus (DM) is a chronic metabolic disease with enormous social, health, and economic consequences [1]. Accordingly, DM belongs to the leading global risks for mortality worldwide, since the disease results in more patients’ deaths than, for instance, HIV/AIDS [1]. DM reduces the ability of the organism to effectively regulate blood glucose levels resulting in the development of diabetes-related complications. The diabetic vascular complications (DVC) are associated with the most important life-threatening ones accompanying DM. Of note, approximately 25% of newly diagnosed type 2 DM patients already suffer from DVC, which probably means that they had undiagnosed DM for at least five years [2]. On the other hand, patients with type 1 DM are frequently diagnosed with the presence of ketoacidosis [3].

The DM, both type 1 and 2, therapy aims to control and restore glucose homoeostasis in both the postprandial and fasting state [4,5]. The first step in the management of type 2 DM represents lifestyle and dietary modifications, which should precede the pharmacological approach [2]. It is already known that the glucose levels can be controlled via various pathways, namely by reducing glucose absorption and hepatic glucose output, enhancing pancreatic insulin secretion, insulin sensitivity, and peripheral glucose utilization [4]. In this context, several types of anti-diabetics have been used in the clinical practice. Oral anti-diabetic agents, used as the treatment of type 2 DM, include biguanides, sulfonylureas, thiazolidinediones, and α-glucosidase inhibitors [6,7]. When oral therapy fails, the treatment is based on the administration of incretin mimetics and their enhancers as well as insulin-like growth factor, and finally on the application of human recombinant insulin, which is the ultimate therapeutic option for type 1 DM [7]. In addition to adverse effects, drug treatments are not always effective in maintaining euglycemia and avoiding late-stage DM complications [8], which are often worse in type 1 DM.

Although known use of plant treatment of DM has been dated already in 1550 BC, anti-diabetic phytotherapy has dramatically been reduced since the introduction of modern pharmacotherapeutics [9]. In our previous study, we standardized the polyphenol content of *Agrimonia eupatoria* L. and *Cynara cardunculus* L. and compared their antioxidant properties. We showed that both *A. eupatoria* and *C. cardunculus* extracts contain nearly 8% of total polyphenols and are able to protect cells and tissues against oxidative damage acting both as radical scavengers as well as by increasing the antioxidant activity [10]. It has also been demonstrated that the *A. eupatoria* water extracts exhibit “insulin-like” activity which also prompted our current study [11,12,13]. Since *C. cardunculus* extracts decrease the postprandial glucose levels in diabetic rats [14,15,16], we set this plant for comparison to the *A. eupatoria* in this study as well.

To evaluate the potential anti-diabetic activity of the plants mentioned above, we determined the inhibitory effect of α-glucosidase and serum glucose levels. To complete the panel of experiments inhibition of advanced glycation end-products (AGEs), the activity of butyrylcholinesterase (BuChE), reactivity of aortas, and measurement of body weight were employed to assess the plants protective properties against the development of DVC.

## 2. Results and Discussion

### 2.1. Inhibition of α-Glucosidase Activity

**Figure 1:**
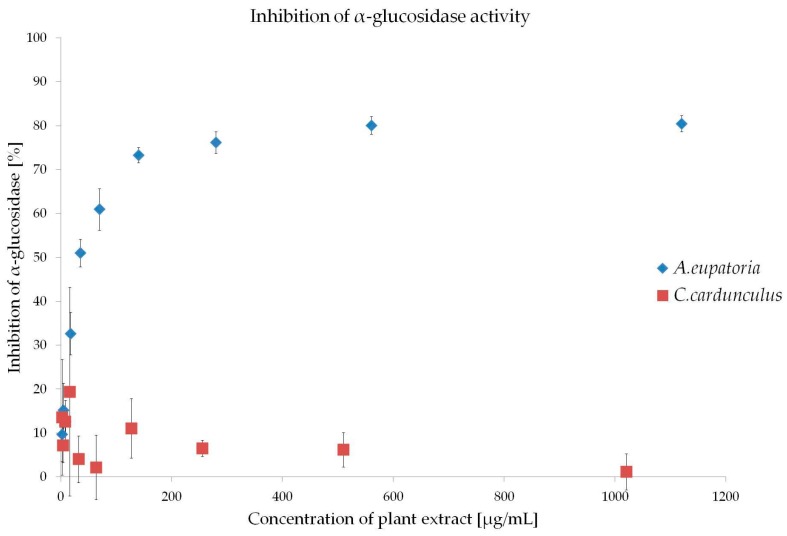
Inhibition of α-glucosidase by *A. eupatoria* (IC_50_ = 46.31 ± 8.76 μg/mL) and *C. cardunculus* (IC_50_ not achieved) water extracts. Data are shown as mean ± SD and were compared using the one-way ANOVA followed by Tukey-Kramer *post-hoc* test.

α-Glucosidase inhibitors have been specifically developed to delay the cleveage of oligo- and polysaccharides to monosaccharides, and thus reduce the postprandial hyperglycemia [6]. Several plants are well known for their α-glucosidase inhibitory activities. In particular, flavonoids and terpenoids have been suggested to be the active compounds responsible for these inhibitory activities [17]. Our results show that *A. eupatoria* water extract is an excellent α-glucosidase inhibitor with IC_50_ = 46.31 ± 8.76 μg/mL (Figure 1), which is in agreement with previously published data [11]. Unfortunately, tested *C. cardunculus* water extract did not exceed 50% of inhibition (Figure 1), thus IC_50_ was not determined. Two hypotheses may explain the observed ineffectiveness of the extract. Firstly, the extraction protocol described in the Pharmacopoeia Bohemoslovaca does not allow us to achieve an efficient compound concentration that is comparable to other studies [15,17]. Secondly, it may also be speculated that the extract compounds inhibiting α-glucosidase activity are not present in tested extracts.

Previously, antioxidant and α-glucosidase inhibition activities of selected compounds isolated from *Agrimonia pilosa* were studied [17]. Here, flavonoid and triterpenoid compounds have shown to be effective inhibitors of α-glucosidase in a dose dependent manner (IC_50_ = 1.67–41.67 μg/mL) [17]. This inhibitory effect of α-glucosidase has also been recorded during several experiments conducted with five different plant extracts from the *Agrimonia* genus (*Agrimonia eupatoria* L., *Agrimonia procera* Wallr., *Agrimonia leucantha* Kze., *Agrimonia japonica* (Miq.) Koidz., and *Agrimonia coreana* Nak.) [11].

From a clinical point of view, the main advantage of daily use of α-glucosidase inhibitors is a relatively low frequency of hypoglycemia [6]. On the other hand, adverse effects of α-glucosidase inhibitors (e.g., acarbose, miglitol) are gastrointestinal complaints, including flatulence and abdominal discomfort, resulting from malabsorption and consequently increased fermentation of carbohydrates [6,7]. However, it is generally accepted that slow dose increase improves the gastrointestinal tolerability.

### 2.2. Inhibition of AGE Formation

**Figure 2:**
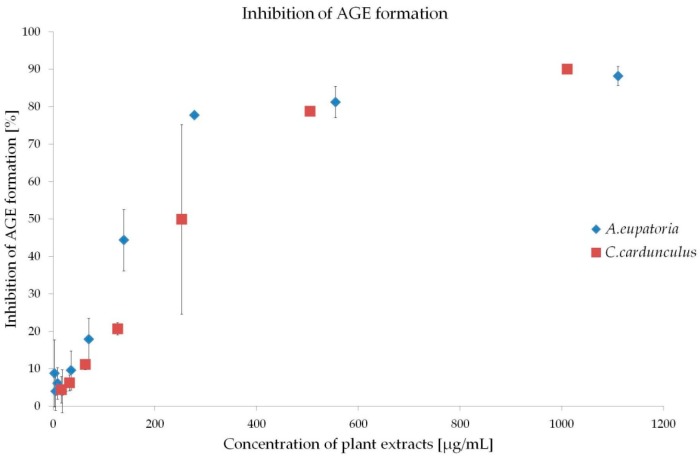
Inhibition of AGE (advanced glycation end-products) formation by *A. eupatoria* and *C. cardunculus* water extracts in the BSA-GLC (bovine serum albumin-glucose) model. Data are shown as mean ± SD and were compared using the one-way ANOVA followed by Tukey-Kramer *post-hoc* test.

The therapeutic strategies preventing the development of DVC include administration of inhibitors of glycation, AGE formation, RAGE (receptors for AGE) activation and protein cross linking inhibitors as well as anti-inflammatory and anti-oxidative agents [4,18]. The present experiment revealed high anti-glycation activity of both extracts in the BSA-GLC (bovine serum albumin-glucose) model (early stage of glycation) (Figure 2). IC_50_ of *A. eupatoria* was determined at 156.48 ± 70.75 μg/mL and that of *C. cardunculus* at 223.61 ± 36.33 μg/mL.

Various plant extracts that were rich in polyphenols have shown antioxidant and anti-glycation activities comparable to *A. eupatoria* and/or *C. cardunculus* [19,20,21]. Previously, we reported high polyphenol content of both water extracts [10], which may represent the way these plants inhibit the development of DVC.

### 2.3. Effect of A. eupatoria and C. cardunculus on Serum Glucose Levels and Body Weights

**Figure 3:**
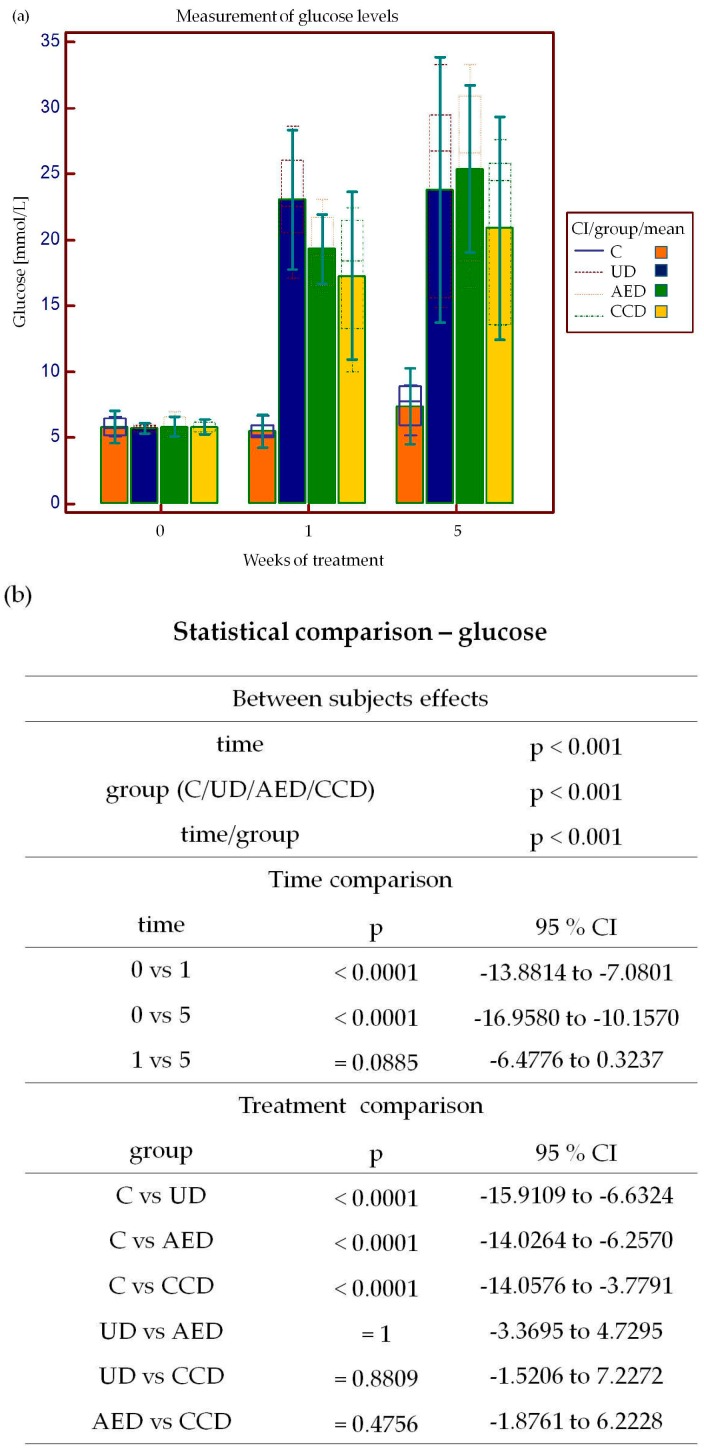

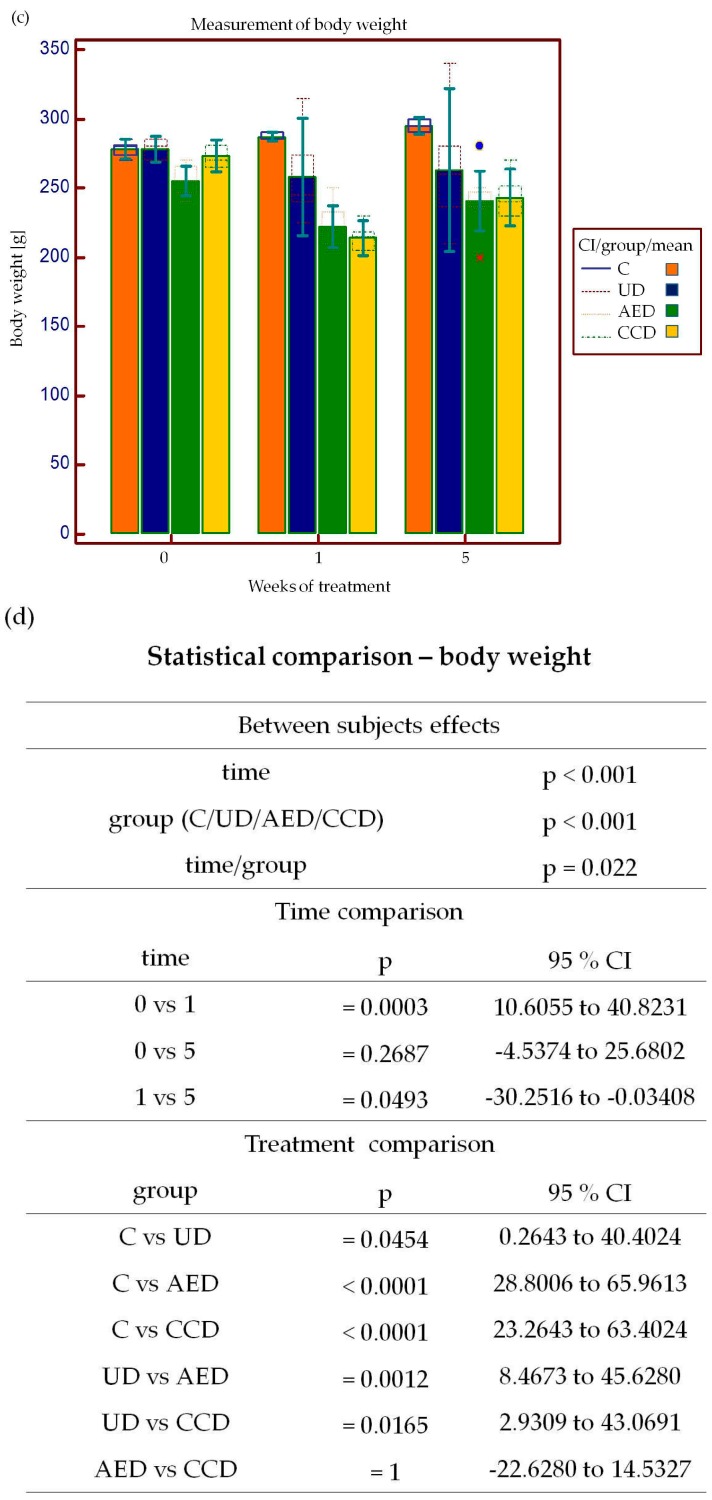
Effect of *A. eupatoria* and *C. cardunculus* water extracts on plasma glucose levels (**a**) and body weight in rats (**c**). Statistical comparison of the glucose levels (**b**) and body weight (**d**) between individual groups (CI—95% confidential interval, C—control group, UD—untreated diabetic group, AED—*A. eupatoria* treated diabetic group, CCD—*C. cardunculus* treated diabetic group). Data are shown as mean ± SD and were compared using the two-way ANOVA followed by Tukey *post-hoc* test.

In the present study, we were interested in whether the extraction protocol described in the Pharmacopoeia Bohemoslovaca [22] exerts significant anti-diabetic properties as described previously [9,12,13,14,15,16,23,24]. We showed that daily oral administration of the *C. cardunculus* extract significantly decreased glycemia after streptozotocin (STZ) administration, but the *A. eupatoria* extract had no anti-glycemic effect (Figure 3a). Although in the present study, the effect of *A. eupatoria* (0.2 mg/mL) was not significant, several studies have proven that the plant’s water extract (1 mg/mL) exerts “insulin-like” activity by at least three potential mechanisms, *i.e.*, stimulation of 2-deoxy-glucose transport, glucose oxidation, and incorporation of glucose into glycogen [12,13]. It has also been shown that the *A. eupatoria* aqueous extract stimulates glucose-independent insulin secretion in BRIN-BD11 pancreatic β-cells [12,13,23,24]. Further examination of the potential mechanisms has also revealed that *A. eupatoria* reduces weight loss, polydipsia, and hyperphagia in diabetic rats [9,12,23,24]. Hence, our results indicate that the extraction procedure used in the present experiment is not sufficient to achieve a required compound concentration with significant anti-diabetic properties.

A further potential mechanism by which *C. cardunculus* may be involved in the regulation of glycemia represents its anti-hyperglycemic effect [14,15,16]. It has been suggested that *C. cardunculus* preparations are capable of reducing fasting and postprandial blood glucose levels in normal and obese rats [14] as well as in selected individuals with type 2 DM [16]. From this point of view, natural remedies containing *C. cardunculus* are recommended as dietary supplements for patients with type 2 DM [16]. However, the exact anti-hyperglycemic mechanism of *C. cardunculus* is still unknown. It may be hypothesized that the high content of roughage in artichokes improves satiety and thus indirectly reduces glycemia [14,15,16,25].

Symptoms of marked hyperglycemia frequently include polyuria, polydipsia, blurred vision, fatigue, nausea, dizziness, impaired wound healing, and weight loss [6]. Since weight loss belongs to typical type-1 DM manifestation [26], the body weight was measured at the beginning of the experiment as well as one and five weeks following STZ administration. Several animal studies have noticed that plant extracts and/or isolated plant compounds are capable of minimizing the body weight loss in the STZ-induced DM model [26,27]. Nevertheless, in the present experiment, long-term administration of neither *A. eupatoria* nor *C. cardunculus* reverses the negative influence of STZ administration on the body weight (Figure 3c).

### 2.4. Determination of BuChE Activity

**Figure 4:**
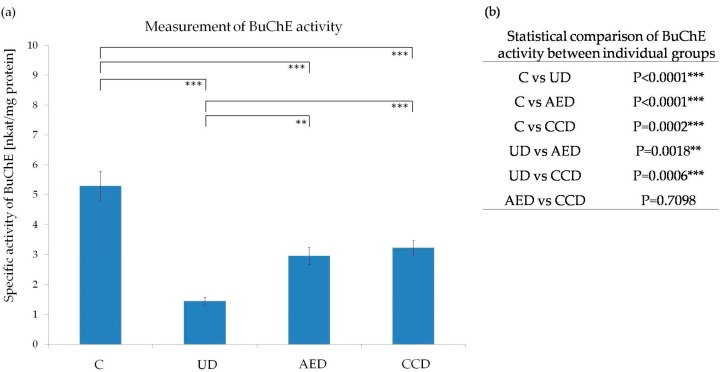
(**a**) Effect of *A. eupatoria* and *C. cardunculus* on specific activity of BuChE in rat livers; (**b**) Statistical comparison of BuChE activity between individual groups (C—control group, UD—untreated diabetic group, AED—*A. eupatoria* treated diabetic group, CCD—*C. cardunculus* treated diabetic group; *** *p* < 0.001, ** *p* < 0.01). Data are shown as mean ± SD and were compared using the one-way ANOVA followed by Tukey-Kramer *post-hoc* test. Differences in the BuChE activity were statistically significant between all groups except AED *vs.* CCD.

It is generally accepted that BuChE has become a therapeutic target and effective marker predicting early development of type 2 DM [28]. Therefore, in the present experiment, the BuChE activity was also determined from each liver sample (Figure 4). The lowest measured activity of BuChE was recorded in the untreated diabetic group. Treatment with both tested plant extracts significantly increased BuChE activity, but it still remained significantly lower than in the control group.

Although the exact roles of BuChE in various physiological and pathological pathways have still remained unclear [29,30], it is already well known that patients with hyperlipidemia, type 2 DM, metabolic syndrome, and/or obesity have elevated levels of BuChE [29,30,31]. Similarly, it has been shown that long-term diabetes alters lipid metabolism with increasing BuChE activity [32]. On the other hand, during the gestational DM, BuChE activity is decreased [33]. Furthermore, a remarkable decrease in the BuChE activity was observed in retinas of STZ-induced diabetic rats [34] and following oxidative stress [35]. In the present experiment, we may consider the STZ-induced type 1 DM as short-term and a stressful event, which may lead to decreased BuChE activity. Notably, enhanced oxidative stress and generation of reactive oxygen species is common in diabetes [4,18,26,27,35,36]. From this point of view, we can hypothesize that *A. eupatoria* and *C. cardunculus* extracts increase BuChE activity in DM rats by their ability to act as hypolipidemic [14,15,16,37], hypocholesterolemic [14,15,16], hepatoprotective [38,39,40,41], and antioxidant [10] agents.

### 2.5. Reactivity of Aortal Rings

**Figure 5:**
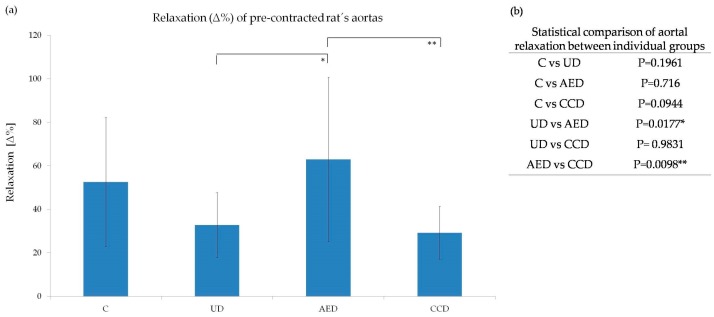
(**a**) Changes in reactibility of a phenylephrine pre-contracted rat’s aorta on acetylcholine (10^−6^ mol/L) after long-treated *A. eupatoria* and/or *C. cardunculus*; (**b**) Statistical comparison of aortal relaxation between individual groups. (C—control group, UD—untreated diabetic group, AED—*A. eupatoria* treated diabetic group, CCD—*C. cardunculus* treated diabetic group; ** *p* < 0.01, * *p* < 0.05). Data are shown as mean ± SD and were compared using the one-way ANOVA followed by Tukey-Kramer *post-hoc* test. Difference in aorta relaxations were statistically significant only between selected groups: UD *vs.* AED and AED *vs.* CCD.

The DVCs include several types of cardiovascular damage, such as peripheral arterial dysfunction, hypertension, endothelial dysfunction, coronary artery blockage, stroke, heart failure, *etc.* [42]. Therefore, in the current study, we addressed the issue of whether *A. eupatoria* and/or *C. cardunculus* water extracts modulate vascular reactivity. The results from our study showed that damage of the endothelium caused by hyperglycemia worsened the dilatatory ability of aortas. However, only treatment with *A. eupatoria* extract significantly improved aorta relaxation, whereas daily administration of *C. cardunculus* extract had no significant effects (Figure 5).

Positive effects of tested extracts observed here may also be related to the high content of polyphenols [10]. These compounds are well known for their endothelium dependent relaxation activities as a result of enhanced nitric oxide (NO) biological activity and/or synthesis [43,44] as well as by protection of NO from breakdown by superoxides [43]. Epicatechin, one of the *A. eupatoria* active compounds [39], may also be responsible for the endothelium-dependent vasorelaxation in STZ-induced diabetic rat arteries by at least two mechanisms—firstly, by enhancing NO biological activity and secondly, by NO-dependent activation of iberiotoxin-sensitive K^+^ channels [45]. Taken together, with enhanced bioavailability of NO, antioxidant [10], anti-inflammatory [37,46,47,48], anti-diabetic, and anti-glycation properties, both extracts exert beneficial effects to prevent the development and progression of DVCs. However, the exact underlying mechanisms of observed protective effects still remain largely unknown.

## 3. Materials and Methods

### 3.1. Plant Material

Throughout the whole experiment, freeze-dried water infusions of *Agrimonia eupatoria* L. and *Cynara cardunculus* L. (both from Fytopharma, Malacky, Slovakia) were used. The water infusions (200 mg/L) were prepared according to Pharmacopoeia Bohemoslovaca 4 [22]. For the *in vivo* study, infusions were prepared fresh each day (200 mg/L). For the *in vitro* experiments, water infusions were subsequently lyophilized (−53 °C, 0.043 Pa) according to the manufacturer’s instructions (SCANVAC CoolSafe™, LaboGene™, Lynge, Denmark).

### 3.2. Inhibition Assay for α-Glucosidase Activity

Briefly, 0.075 units of α-glucosidase (Sigma-Aldrich, St. Louis, MO, USA), from *Saccharomyces cerevisae*, were premixed with the extracts at various concentrations (0.5–1000 μg/mL). To start the reaction, 3 mM *p*-nitrophenyl α-glucopyranoside (*p*NPG) (Sigma-Aldrich) in sodium phosphate buffer (pH 7.4) was added to the mixture as a substrate. The reaction was incubated at 37 °C for 30 min and stopped by adding 2 mL of 0.1 M Na_2_CO_3_ (Sigma-Aldrich). The α-glucosidase activity was determined by measuring the *p*-nitrophenol release from *p*NPG at 400 nm using a plate reader (TECAN Infinite^®^ M 200, Männedorf, Switzerland). The IC_50_ value was defined as the concentration of α-glucosidase inhibitor used to inhibit 50% of its activity under the assay conditions [49].

### 3.3. Inhibition of AGE Formation (Glycation)—BSA-Glucose Assay

For the determination of anti-glycation activity, the BSA-GLC assay was used [50,51]. Briefly, 1 g of bovine serum albumin (BSA, Sigma-Aldrich) was dissolved in 50 mL sodium phosphate buffer (pH 7.4), and 10 g glucose (Sigma-Aldrich) was dissolved in 50 mL of distilled water. The test solutions contained 0.5 mL of plant extract (in different concentrations: 0.5–1000 μg/mL) or distilled water (control), 0.75 mL BSA solution, and 0.75 mL glucose solution. These solutions were incubated at 37 °C for 7 days. After the incubation, fluorescent intensity (excitation = 330 nm, emission = 410 nm) was measured on the plate reader (TECAN, Infinite^®^ M 200, Männedorf, Switzerland). The percentage of inhibition of AGE formation was calculated using the following equation:

% inhibition = (1 − fluorescence of the solution with inhibitors/fluorescence of the solution without inhibitors (control)) × 100.
(1)

### 3.4. Animal Model

The animal experiment was approved by the Ethical Committee of Faculty of Pharmacy, Comenius University in Bratislava (no. 962/10-221). Twenty-four male Wistar rats (DobráVoda, Slovak Republic) weighing 278 ± 5.70 g were used in the experiment. After a brief acclimatization period, rats were randomly divided into 4 groups (6 rats per group): (1) healthy animals—control group; (2) untreated diabetic group (DM was induced by *i.p.* (intraperitoneal) administration of STZ (Sigma-Aldrich) diluted in citrate buffer (pH 4.5) at a dose of 55 mg/kg); (3) and (4) diabetic animals daily treated for 5 weeks with tested plant extracts. Prior STZ administration blood glucose levels and total body weight of all animals were measured. The blood glucose levels and body weight were subsequently measured 1 and 5 weeks after DM induction.

Following a 5-week period, all rats were killed by anesthetic overdose (thiopental, 100 mg/kg *i.p.*, Sigma-Aldrich). Thoracic aortas and livers were immediately removed, repeatedly rinsed in saline, frozen in liquid nitrogen, and stored at −80 °C until the analysis.

### 3.5. Preparation of Liver Homogenates

The tissue homogenates were prepared by intensive homogenization of 0.5 g of tissue and 10 mL of cold potassium PBS (pH = 7.4, with addition of 0.5% Triton™X (Sigma-Aldrich)). The tissue homogenates were then centrifuged at 19,872 *g* for 15 min at 4 °C. The supernatant was subsequently collected and used for analysis. The protein content was determined according to Bradford protocol [52].

### 3.6. Determination of BuChE Activity

For the determination of BuChE activity, the Ellman method was used [53]. Briefly, the reaction mixture contained 1 mL of 5,5′-dithiobis(2-nitrobenzoic) acid (DTNB, Sigma-Aldrich), 0.03 mL of tissue homogenate from livers and 0.370 mL of potassium phosphate buffer (pH = 7.4).The reaction took place in a silicon cuvette (d = 1 cm). Following a 20-minutepreincubation period at room temperature, 0.1 mL of butyrylthiocholine (Sigma-Aldrich) was added into the cuvette. The intensity of the end-product 5-thio-2-nitrobenzoic acid (TNB) was spectrophotometrically measured at 412 nm (Genesys 6, Thermo, Electro Corp., Loughborough, UK) in 5-minute intervals during a 25 min period. The specific activity of BeChE was then calculated from the following equation:

nkat = A × V_im_ × 1000/ε × d × t × V_e_ (2)

and expressed as nkat/mg of proteins (A—absorbance; V_im_—total volume of the incubation mixture (1.5 mL); ε—molar absorption coefficient of TNB (13.6 mmol^−1^·cm^−1^); d—thickness of the cuvette; t—time; V_e_—volume of the enzyme).

### 3.7. Isolation and Reactivity Evaluation of Aortal Rings

Immediately after euthanasia of animals, aortas were removed, cut into rings, and placed between parallel wires in a tissue bath at 37 °C, which contained Krebs-Henseleit solution gassed with 95% O_2_ and 5% CO_2_. Rings from all rats were stretched to an optimal resting tension of 1.5 g and stabilized for 60 min. The aortal rings were pre-contracted with 10^−6^ mol/L of phenylephrine (Sigma-Aldrich), to achieve the maximal response of aortas. Subsequently, acetylcholine (Sigma-Aldrich) in submaximal concentration of 10^−6^ mol/L was applied. Changes in tension were isometrically monitored and recorded using a S.P.E.L. Advanced Isosys Software (Experimetria Ltd., Budapest, Hungary). Modulatory effect of the extracts were expressed as a maximal percentage of the relaxation (∆%).

### 3.8. Statistical Analysis

Data from the experiments are present as mean ± standard deviation (SD). One-way analysis of variance (ANOVA) followed by Tukey-Kramer *post-hoc* test were used to compare the differences between selected groups (*i.e.*, inhibition of α-glucosidase and AGE formation, determination of BuChE activity, and reactivity of aortal rings). To compare the glucose levels and body weights, two-way analysis of variance (ANOVA) followed by Tukey *post-hoc* test were used. Significance was accepted at *p* < 0.05.

## 4. Conclusions

Results obtained in the present paper indicate that both *A. eupatoria* and *C. cardunculus* extracts exert several anti-diabetic activities. Small differences in the obtained results may be related to diverse phytochemical composition of tested extracts [10]. *A. eupatoria* was characterized mainly by procyanidins and flavonol glycosides and *C. cardunculus* by chlorogenic acid derivatives [10]. In general, *A. eupatoria* and *C. cardunculus* demonstrated good anti-glucosidase, anti-glycation, and anti-hyperglycemic effects. Furthermore, the experiments on isolated aortas showed improvement of vascular dilatatory functions in diabetic animals. However, higher anti-oxidant activity of the *A. eupatoria* extract [10] indicates its better clinical potential in the prevention and/or adjuvant therapy of developing cardiovascular complications related to diabetes and diseases associated with oxidative stress. Nevertheless, the optimal treatment protocol for use in humans remains to be found in further clinical studies.
